# Outcomes of Cardiopulmonary Resuscitation in Patients Admitted to the Intensive Care Unit: A Single-Center, Retrospective Study

**DOI:** 10.7759/cureus.107349

**Published:** 2026-04-19

**Authors:** Yogesh Manhas, Manish Kumar Tiwary, Sulaiman Al Rahbi, Abhijit Nair

**Affiliations:** 1 Critical Care, Ibra Hospital, Ibra, OMN; 2 Anesthesiology, Sheikh Khalifa Medical City, Ajman, ARE; 3 Anesthesiology, Ibra Hospital, Ibra, OMN

**Keywords:** cardiac arrest outcome, cardiopulmonary resuscitation, do-not-resuscitate (dnr) status, in-hospital cardiac arrest, return of spontaneous circulation (rosc)

## Abstract

Background: The study was performed to evaluate the outcomes of cardiopulmonary resuscitation (CPR) among patients who suffered cardiac arrest in the intensive care unit (ICU).

Methods: We carried out a single-center study. Retrospective hospital records were reviewed for patients admitted to the ICU from January 2020 to May 2022. Data included patient demographics, reason for ICU admission, presence of comorbidities, rhythm at arrest, achievement of return of spontaneous circulation, number of CPR attempts, cause of arrest, use of inotropes/vasopressors before arrest, do-not-resuscitate (DNR) status, and survival rate.

Results: A total of 544 patients were admitted to the ICU during the study period. Seventy-five patients suffered cardiac arrest. CPR was conducted in 59 patients. Sixteen patients were declared DNR before cardiac arrest occurred. Only one patient survived among those who received CPR. Quantitative data were expressed in numbers, while the Mid-P exact test was the test of significance to determine the relationship between the use of inotropes/vasopressors before arrest and survival of the patients.

Conclusions: Of 59 patients who received CPR, only one patient (1.7%) made it to hospital discharge, indicating a very poor survival rate for critically ill patients.

## Introduction

Cardiopulmonary resuscitation (CPR) is a life-saving procedure. It has become a default practice in all scenarios where cardiorespiratory arrest occurs, though originally it was intended for those who experience a sudden and unexpected cardiac arrest with a presumed high chance of functional recovery [1]. As a result, CPR performed in critically ill patients already dependent on life support measures is associated with poor survival and functional outcomes [2].

Continuing life support and attempts at CPR where chances of survival are poor not only prolong the suffering of the patients but also increase the workload of the staff, burdening intensive care unit (ICU) resources and imposing financial stress [3,4]. The prevalence of cardiac arrest in the ICU has been reported to be 0.5%-7.8% in various studies [5]. The success rate of CPR is reported to range from 3.1% to 16.5% [6-8]. The objective of the study is to know the outcome of the CPR in critically ill patients, the characteristics of the patients who do not survive the CPR attempt, while also examining the context in which do-not-resuscitate (DNR) decisions were made.

## Materials and methods

This study was conducted at Ibra Hospital, a secondary care hospital under the Ministry of Health, located in North Sharqiya Governorate of the Sultanate of Oman. Approval was obtained from the Center of Studies and Research, the Directorate General of Planning and Studies, and the Ministry of Health, Oman. Data were retrieved from hospital electronic records (Al-Shifa system) from January 2020 to May 2022, with the help of an IT professional, using the keywords "cardiopulmonary resuscitation (CPR)", "cardiac arrest", "return of spontaneous circulation (ROSC)", and "do not resuscitate (DNR)". To avoid missing information about any patient, we manually searched the electronic records for the period mentioned above.

Patients who suffered cardiac arrest outside the ICU and pediatric patients aged less than 12 years were excluded. Data included patient demographics, reason for ICU admission, presence of comorbidities, rhythm at arrest, achievement of return of spontaneous circulation (ROSC), number of CPR attempts, cause of arrest, use of inotropes/vasopressors before arrest, DNR status, and survival rate. The data thus collected were compiled, entered into a Microsoft Excel sheet (Microsoft Corporation, Redmond, WA), tabulated, and analyzed. Quantitative data were expressed as numbers, while the Mid-P exact test was used to determine the relationship between the requirement for vasopressors/inotropes before cardiac arrest and patient survival, using the OpenEpi software version 3.01 (Dean AG, Sullivan KM, Soe MM. OpenEpi: Open Source Epidemiologic Statistics for Public Health, Version. www.OpenEpi.com, updated 2013/04/06). Given the small sample size, the Mid‑P exact test was chosen after consultation with a senior biostatistician, as it provides more reliable estimates than the traditional Fisher’s exact test in small datasets. A p value of <0.05 was considered statistically significant.

## Results

During the studied period, 544 patients were admitted to the ICU. A total of 75 patients suffered cardiac arrest. Forty-seven were male and 28 were female patients. Demographic details like gender, comorbidities, reason for ICU admission, and possible cause of cardiac arrest are summarized in Table 1. Pulseless electrical activity (PEA) and asystole were the rhythms associated with cardiac arrest.

**Table 1:** Summary of demography, comorbidities, reason for ICU admission, and cause of cardiac arrest ICU: intensive care unit; COPD: chronic obstructive pulmonary disease; ARDS: acute respiratory distress syndrome

Variables	n (%)
Gender	
Male	47 (62.6)
Female	28 (37.3)
Comorbidities	
Hypertension	43 (57)
Diabetes mellitus	37 (49)
Coronary artery disease	25 (33)
Heart failure	21 (21)
Chronic kidney disease	11 (14)
COPD	13 (17)
Liver disease	2 (2.6)
Reason for ICU admission	
Pneumonia	11 (14)
Sepsis other than pneumonia	17 (22)
Cerebrovascular accident	11 (14)
Trauma	9 (12)
ARDS	9 (12)
Heart failure	6 (8)
Acute coronary syndrome	3 (4)
Hemorrhage	2 (2.6)
Intestinal perforation	2 (2.6)
Asthma	1 (1.3)
Obstructed hernia	1 (1.3)
Pulmonary embolism	1 (1.3)
Acute disseminated encephalomyelitis	1 (1.3)
Postcardiac arrest	1 (1.3)
Cause of cardiac arrest	
Acidosis	11 (14.6)
Hyperkalemia	3 (4)
Hypoxia	3 (4)
Hemorrhage	2 (2.6)
Myocardial infarction	1 (1.3)
Pulmonary embolism	1 (1.3)
Pneumothorax	1 (1.3)
Unknown	53 (70.6)

**Figure 1:**
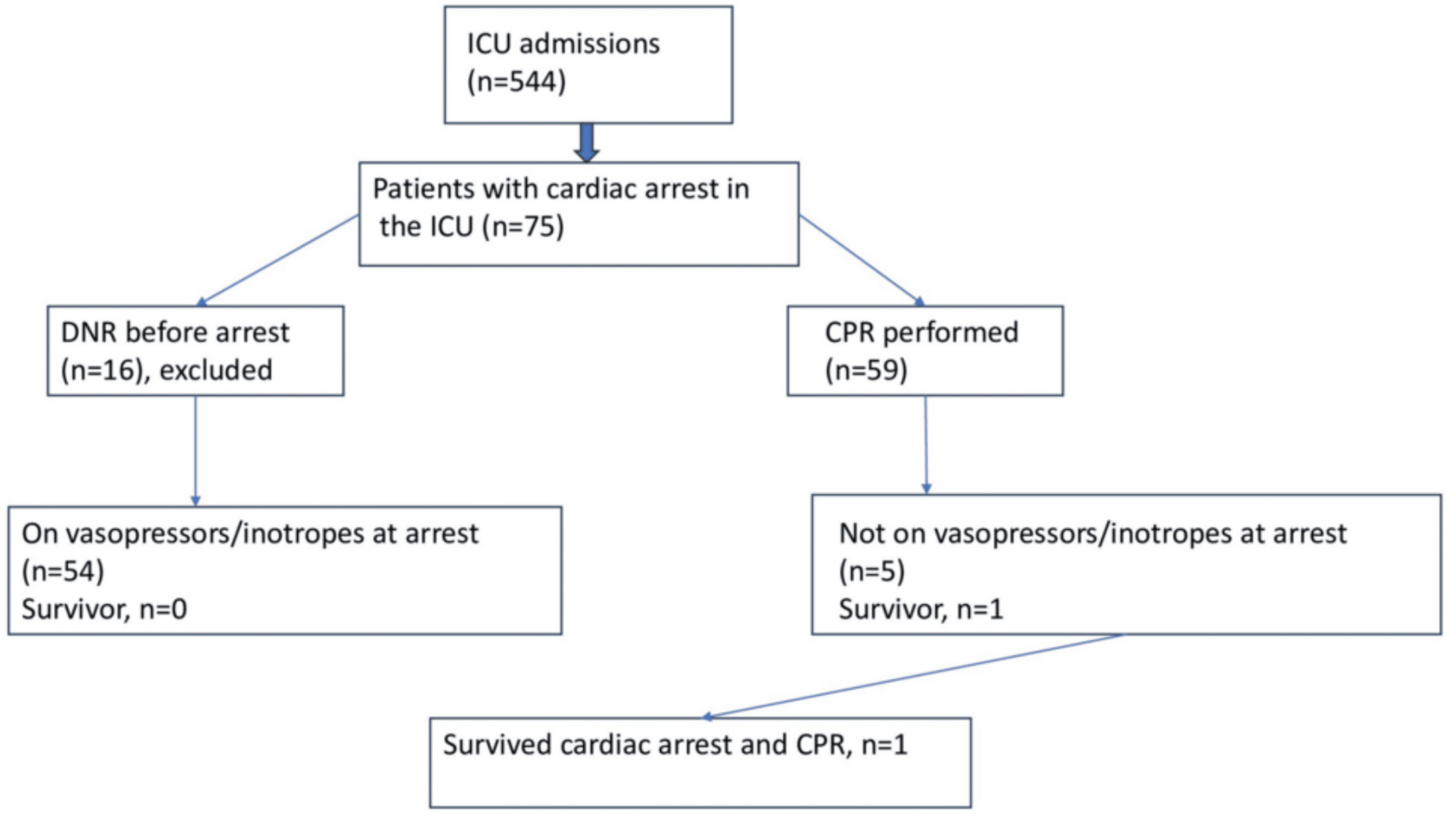
Flowchart summarizing the cardiac arrest, CPR, and outcomes of patients admitted to the ICU ICU: intensive care unit; DNR: do not resuscitate; CPR; cardiopulmonary resuscitation

Sixteen patients were coded DNR; thus, CPR was performed on 59 patients. The DNR status was decided after a comprehensive discussion with the family members, after explaining to them the prognosis of the patient in detail. Only one of 59 patients who received CPR survived. However, ROSC was initially achieved in 57% of patients, but they subsequently experienced arrests and were not revived. In the majority of patients (70.6%), the cause of cardiac arrest could not be ascertained. Several patients underwent CPR multiple times, some as many as six times over a short period of time (Figure 1).

Fourteen patients were coded DNR after multiple CPR attempts. Sixty-seven (89%) patients were receiving vasopressors/inotropes when cardiac arrest occurred (Table 2).

**Table 2:** Details of patient survival based on the use of vasopressors/inotropes

Parameter	Patients survived	Patients not survived	Total	p value
Vasopressor/inotropes used	0	54	54	p value (one-tailed) = 0.04; mid-P-exact: p value (two-tailed) = 0.08
No use of vasopressor/inotropes	1	4	5	
Total	1	58	59	

## Discussion

The study showed a poor post-CPR survival among critically ill patients who suffered cardiac arrest in the ICU. Only one patient (1.69%) survived out of 59. Although we did not measure illness severity, it is possible that patients in our study were too ill to be considered for DNR status. In fact, this study was undertaken based on an observation that there was a reluctance in discussing DNR code status with patient families.

ROSC was achieved in 57% of the patients, which is consistent with the results of a large study, though patients in our study had subsequent cardiac arrest and did not survive [9]. Our study found an association between vasopressors/inotropes before cardiac arrest and poor survival. None of the patients requiring vasopressors/inotropes survived. Previously, studies have also reported poor survival among patients who required vasopressors before cardiac arrest occurred [10,11].

One significant predictor of outcome is the rhythm at the time of cardiac arrest. The vast majority of in-hospital cardiac arrests (IHCAs) are caused by nonshockable rhythms, which include asystole and PEA, accounting for about 81% of presenting rhythms according to a United States registry data. Recent research indicates that these two rhythms are separate clinical entities, even though they are treated using the same advanced cardiovascular life support algorithm. While asystole typically occurs in unmonitored ward settings, PEA arrests are more common in the ICU, often in patients already on mechanical ventilation or vasopressors [12]. Several predictors determine poor outcomes after ROSC in IHCA. These are associated comorbidities (two or more), longer CPR duration (more than 20 minutes), lower Glasgow Coma Scale (less than 3), higher lactate levels (more than 4 mmol/L), and lower mean arterial pressure (less than 65 mmHg) [13,14]. The complexity of the critically ill ICU population, where numerous interacting pathologies and associated comorbidities make it challenging to identify a single precipitating event, is reflected in our study, where in the majority of patients (70.6%), we were unable to determine the cause of arrest.

The role of early and transparent discussions around DNR status has been emphasized, but it is equally challenging because it requires a balanced medical judgment, patient autonomy, family expectations, and ethical concerns. In our study, 14 patients were assigned DNR status only after multiple CPR attempts had already been made, highlighting a pattern of delayed code status activation [15]. Alahmadi et al. conducted a cross-sectional study to investigate DNR order awareness and perceptions among physicians in Saudi Arabia and other Middle East countries. The results of this study revealed that patient and family misunderstandings were the main barriers to DNR implementation. Another barrier was the lack of training and understanding of the concept of DNR orders among the physicians who participated in the survey [16].

Life-sustaining treatments also increase the probability of nonbeneficial medical treatments being given to patients at the end of life. Therefore, an early documented DNR order is essential, based on the clinician's assessment of the sick patient, the involvement of family members in shared decision-making, and adherence to institutional protocol in such circumstances [17].

Our study was limited by its retrospective design and a small sample size. In addition, we did not measure other variables that would have been more informative, such as the severity of illness and the need for mechanical ventilation. We also did not quantify in the results the number/percentages of the rhythm detected at cardiac arrest. Our analysis was limited to examining the association between vasopressor/inotrope use and survival, as other important predictors, such as severity-of-illness scores and rhythm-specific outcomes, were not consistently available in the retrospective data gathered. Thus, the results of our study should be interpreted with caution. Nonetheless, the study does highlight the importance of assessing the futility of CPR in critically ill patients who are on life support measures promptly. Predictive models such as the Pre-Arrest Morbidity score and the Good Outcome Following Attempted Resuscitation score are used to predict post-CPR outcomes [18,19]. Though this score predicts postcardiac arrest mortality with reasonable accuracy, it is not specific to critically ill patients, as the study included all IHCAs [20]. Nevertheless, our research sheds light on local CPR results and emphasizes the critical need for improved prognostic evaluation and DNR implementation techniques in ICUs.

Future studies are needed to develop predictive tools to determine the futility of CPR specifically for the ICU patient population. Prospective, multicenter studies with larger sample sizes, standardized documentation of prearrest variables, and validated severity scoring systems should be the focus of future research.

## Conclusions

Only one patient of 59 who received CPR survived to be discharged from the hospital, indicating a very poor survival rate for critically ill patients. Given that the majority of the patients were on life-sustaining measures and had severe illnesses, these results indicate the limited efficacy of CPR in patients who had already been admitted to the ICU.
